# Altered synchronous neural activities in retinal vein occlusion patients: A resting-state fMRI study

**DOI:** 10.3389/fnhum.2022.961972

**Published:** 2022-09-16

**Authors:** Yu Mei Xiao, Fan Gan, Hui Liu, Yu Lin Zhong

**Affiliations:** ^1^Department of Operation, Jiangxi Provincial People’s Hospital, The First Affiliated Hospital of Nanchang Medical College, Nanchang, China; ^2^Department of Ophthalmology, Jiangxi Provincial People’s Hospital, The First Affiliated Hospital of Nanchang Medical College, Nanchang, China

**Keywords:** retinal vein occlusion, regional homogeneity, resting-state fMRI, brain activity, fMRI

## Abstract

**Objective:**

Retinal vein occlusion (RVO) is the second most common retinal vascular disorder after diabetic retinopathy, which is the main cause of vision loss. Retinal vein occlusion might lead to macular edema, causing severe vision loss. Previous neuroimaging studies of patients with RVO demonstrated that RVO was accompanied by cerebral changes, and was related to stroke. The purpose of the study is to investigate synchronous neural activity changes in patients with RVO.

**Methods:**

A total of 50 patients with RVO and 48 healthy subjects with matched sex, age, and education were enrolled in the study. The ReHo method was applied to investigate synchronous neural activity changes in patients with RVO.

**Results:**

Compared with HC, patients with RVO showed increased ReHo values in the bilateral cerebellum_4_5. On the contrary, patients with RVO had decreased ReHo values in the bilateral middle occipital gyrus, right cerebelum_crus1, and right inferior temporal gyrus.

**Conclusion:**

Our study demonstrated that patients with RVO were associated with abnormal synchronous neural activities in the cerebellum, middle occipital gyrus, and inferior temporal gyrus. These findings shed new insight into neural mechanisms of vision loss in patients with RVO.

## Introduction

Retinal vein occlusion (RVO) is a common ophthalmic disease, which is the second most common retinal vascular disorder after diabetic retinopathy and is the main cause of vision loss. Retinal vein occlusion can be classified as hemorrhagic. In recent years, growing evidences have demonstrated that there are several risk factors for the occurrence of RVO such as hypertension, (Ponto et al., 2019) hyperlipidemia, (Kolar, 2014) and/or diabetes mellitus (Chang et al., 2021). The clinical manifestations of retinal vein occlusion are flame hemorrhages, dot and blot hemorrhages, cotton wool spots, hard exudates, retinal edema, and dilated tortuous veins (Jaulim et al., 2013). At present, the effective treatments of RVO mainly include injection of anti-VEGF drugs and retinal laser (Ciulla et al., 2021). However, there are still some patients with RVO with poor outcomes and recurrent macular edema. Recent studies have shown that RVO in patients, not only causes changes in the retina, but may also be accompanied by abnormalities in cerebral blood vessels (Park et al., 2015; Rim et al., 2015). Meanwhile, the RVO can be seen as a sign of cerebral disease. Nam et al. (2021) found that the patients with RVO had increased risks of dementia and Alzheimer’s disease. Lee et al. (2021) demonstrated that patients with RVO with APOEε4 allele are at higher risk for vascular dementia. However, at present, recent studies showed the cerebral disease caused by RVO are all indirect evidence and lack direct evidence. The exact pathological mechanism of brain changes caused by RVO is not clear.

Magnetic resonance imaging (MRI) provides us with an important method to assess brain function and structural changes *in vivo*. Dong et al. (2021) reported that patients with RVO showed abnormal brain network hubs related to the right superior parietal lobule, middle frontal gyrus, and left precuneus. Su et al. (2020) found that patients with RVO had abnormal functional connectivity between the primary visual cortex, and visual-related and cognitive-related region. Cho et al. (2017) demonstrated that incidence of cerebral small vessel disease were higher in young patients with RVO. Zhang et al. (2022) reported that patients with RVO were associated with abnormal white matter bundle, which is located in the bilateral posterior thalamic, bilateral sagittal stratum. However, there are few studies on the cerebral functions and structure changes in patients with RVO. The exact synchronous neural activity changes in the patients with RVO remain unknown.

The human brain had synchronized neural activity at rest. The synchronized neural activity plays a critical role in the multiple neurophysiological functions. Piazza et al. (2021) demonstrated that child participants showed synchronized neural activity during book reading and showed a positive correlation between learning and intersubject neural synchronization in the parietal cortex. Grover et al. (2021) also demonstrated that synchronous electrophysiological rhythms are a core mechanism for cognitive function and its breakdown in neuropsychiatric disorders. Benedetto et al. (2021) reported that a synchronized neural rhythm will modulate cortical excitability rhythmically, which should be reflected in sensorimotor and visual areas coherence to encode sensory-motor timing. Regional homogeneity (ReHo) is a novel fMRI method, which is applied to calculate the coherence of blood oxygen levels depend on (BOLD) signals (Zang et al., 2004). The ReHo values are reflected in the cerebral synchronized neural activity changes. The ReHo method has a high temporal and spatial resolution. Besides, the ReHo method is a data-driven technology, which can reflect the whole brain synchronized neural activity changes without beforehand region of interest. The ReHo method has been successfully applied to study the changes in the brain neural mechanisms in various neurological diseases such as depression, (Yan et al., 2021) mild cognitive impairment (Liu et al., 2021) and stroke (Zhao et al., 2018). However, the whole brain synchronized neural activity changes in patients with RVO remain unclear.

Based on these assumptions, our study is to determine whether the patients with RVO were associated with the whole brain synchronized neural activity changes. Our study will shed new light on the neural mechanism of visual loss in patients with RVO.

## Materials and methods

### Participants

In total, fifty patients with RVO (25 men, 25 women, 35 BRVO, and 15 RVO) and 48 HCs (24 men, 24 women) participated in the study. The diagnostic criteria of RVO were by fundus examination such as intraretinal hemorrhages, cotton wool spots, and vascular congestion.

The exclusion criteria of RVO in the study were: (1) RVO accompanied by arterial occlusion or ocular ischemic syndrome; and (2) with other eye diseases (glaucoma, amblyopia, high myopia, or optic neuritis).

All the HCs met the following criteria: (1) no ocular diseases (myopia, cataracts, glaucoma, optic neuritis, or retinal degeneration); (2) binocular visual acuity 1.0; (3) no ocular surgical history; and (4) no mental disorders.

Ethical statement: All the research methods followed the Declaration of Helsinki and were approved by the Ethical Committee for Medicine of Jiangxi Provincial People’s Hospital.

### Clinical data analysis

The visual acuity of all subjects was measured using the logMAR table and intraocular pressure was assessed by automatic intraocular pressure measurement. The best-corrected VA and intraocular pressure of both eyes were measured in each group.

### MRI data acquisition

The MRI scanning was performed on a 3-Tesla MR scanner (750W GE Healthcare, Milwaukee, WI, United States) with an eight-channel head coil. All the participants were required to close their eyes without falling asleep when undergoing MRI scanning. In total, 240 functional images parameters (repetition time = 2,000 ms, echo time = 25 ms, thickness = 3.0 mm, gap = 1.2 mm, acquisition matrix = 64 × 64, field of view = 240 × 240 mm^2^, flip angle = 90°, voxel size = 3.6 × 3.6 × 3.6 mm^3^, and 35 axial slices) covering the whole brain were obtained.

### Data preprocessing

All the preprocessing was performed using the toolbox for Data Processing and Analysis of Brain Imaging (DPABI^1^) (Yan et al., 2016) and specific analysis steps were referred to previous researches (Chen et al., 2022; Wen et al., 2022). The details steps were as follows:(1) Remove first 10-time points of each subject. (2) Slice timing effects, motion corrected, and realigned. (3) Individual T1-weighted images were registered to the mean fMRI data. (4) Normalized to the standard Montreal Neurological Institute (MNI) space. (5) Detrend of the time course was performed. (6) Linear regression analysis was used to remove nuisance covariates (such as white matter signal, six head motion parameters, and cerebrospinal fluid signal). (7) After that, the fMRI images were band pass-filtered (0.01–0.08 Hz) to reduce the effects of low-frequency drift and high-frequency signals.

### ReHo analysis

The ReHo index was calculated by DPABI software. All ReHo maps of each voxel were *z-*transformed with Fisher’s *r-to-z* transformation to reduce the influence of individual variation for group statistical comparisons.

### Statistical analysis

The independent sample *t-*test was used to investigate the clinical features of the two groups.

The one-sample *t-*test was conducted to assess the group mean of ReHo maps. The two-sample *t-*test was used to compare the two group differences in the ReHo and FC maps using the Gaussian random field (GRF) method (two-tailed, voxel-level *P* < 0.05, GRF correction, cluster-level *P* < 0.05).

## Results

### Demographics and disease characteristics

**FIGURE 1:**
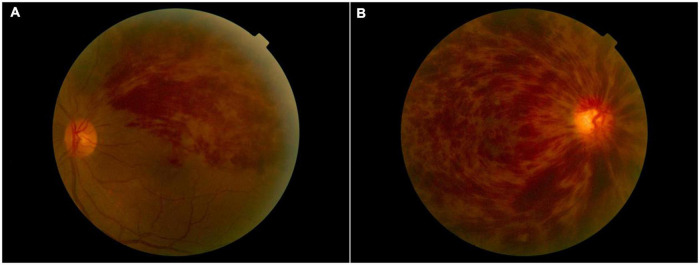
A typical fundus photograph of BRVO **(A)** and CRVO **(B)**. BRVO, branch retinal vein occlusion; CRVO, central retinal vein occlusion.

There were no statistically significant differences between the RVO and HC groups in gender, education, or age, but significant differences in BCVA of the right eye (*p* < 0.001), left eye (*p* < 0.001). The results of these data were listed in Table 1. A typical fundus photograph of BRVO (Figure 1A) and CRVO (Figure 1B).

**TABLE 1:** Clinical information for two groups.

	RVO group	HC group	*T*-values	*P*-values
Sex (male/female)	50	48	N/A	0.780
Mean age (years)	45.15 ± 14.95	45.30 ± 13.87	−0.038	0.970
Education (years)	10.96 ± 3.73	11.12 ± 2.86	−0.164	0.871
BCVA-OD	0.44 ± 0.27	1.16 ± 0.16	−11.474	< 0.001*
BCVA-OS	0.43 ± 0.37	1.19 ± 0.16	−9.352	< 0.001*

Chi-square test for sex.

Independent t-test was used for other normally distributed continuous data.

Data are presented as mean ± standard deviation.

RVO, retinal vein occlusion; HC, healthy control; BCVA, best-corrected visual acuity; OD, oculus dexter; OS, oculus sinister.

*p < 0.05.

### Comparisons of ReHo between patients with PACG and HC

**FIGURE 2:**
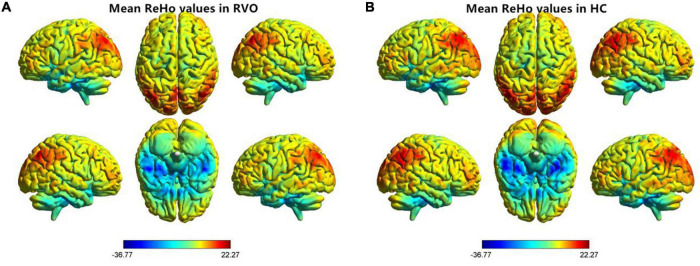
Distribution patterns of the ReHo value at the group level in patients with RVO and HCs. One-sample *t-*test result of ReHo maps within the RVO **(A)** and HCs **(B)**. The color bar represents the *t* values. RVO, retinal vein occlusion; HC, health controls; ReHo, regional homogeneity; L, left; R, right.

**FIGURE 3:**
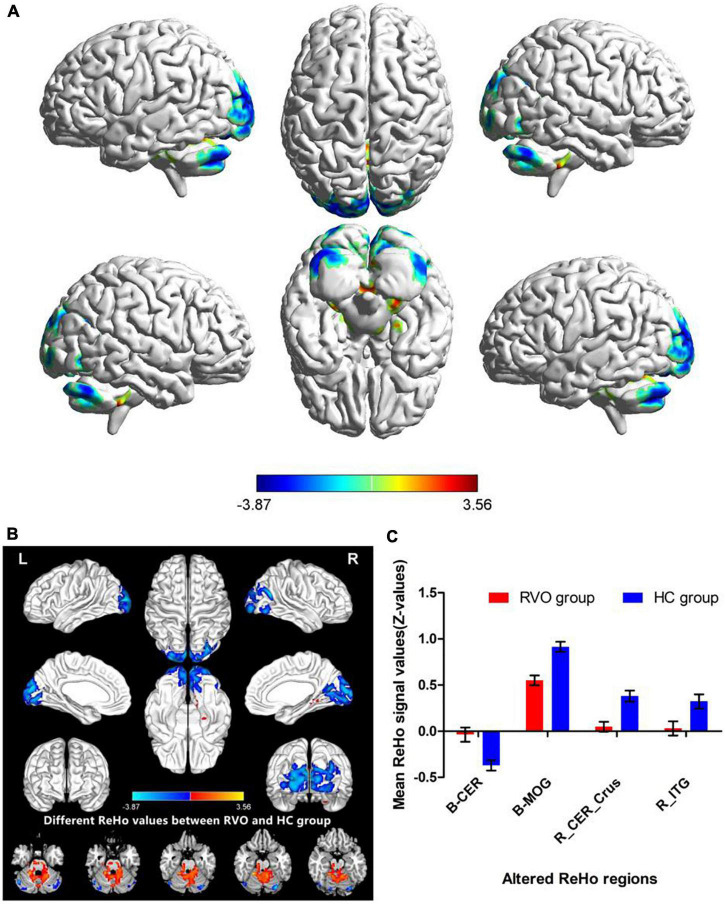
Different ReHo values between RVO group and HC group. **(A)** The 3D picture of different ReHo values between two groups. **(B)** The 2D picture of different ReHo values between two groups. **(C)** The mean values of altered ReHo values were shown with a histogram.

The group means of ReHo maps of the RVO and HC are shown in Figures 2A,B. Compared with HC, patients with RVO showed increased ReHo values in the bilateral cerebellum_4_5. On the contrary, patients with RVO had decreased ReHo values in the bilateral middle occipital gyrus, right cerebellum_crus1, and right inferior temporal gyrus (Table 2 and Figures 3A,B)The mean values of altered ReHo values were shown with a histogram (Figure 3C).

**TABLE 2:** Brain regions with significantly different ReHo signal values between two groups.

Conditions	Brain regions	Cluster size	MNI coordinates			t-score of peak voxel
			X	Y	Z	
RVO > HCs	B-Cerebelum_4_5	1128	27	−27	−51	3.5638
RVO < HCs	B-Middle Occipital Gyrus	1718	3	−99	3	−3.8721
RVO < HCs	R_Cerebelum_Crus1	203	48	−63	−30	−3.4208
RVO < HCs	R_Inferior Temporal Gyrus	26	48	−69	−6	−2.5795

x, y, and z are the locations of the peak voxels in standard MNI coordinates.

The statistical threshold was set at (two-tailed, voxel-level *P* < 0.05, GRF correction, cluster-level *P* < 0.05).

RVO, retinal vein occlusion; ReHo, regional homogeneity; HC, healthy control; MNI, Montreal Neurological Institute; GRF, Gaussian random field; B, bilateral; R, right.

## Discussion

In our study, compared with HC, patients with RVO showed increased ReHo values in the bilateral cerebellum_4_5. On the contrary, patients with RVO had decreased ReHo values in the bilateral middle occipital gyrus, right cerebelum_crus1, and right inferior temporal gyrus. Thus, the patients with RVO were associated with widespread cerebral neural activity changes related to the cerebellum, occipital gyrus, and temporal gyrus.

Our study demonstrated that patients with RVO showed a significant decrease in ReHo values in the bilateral middle occipital gyrus. The middle occipital gyrus is the location of the primary visual cortex, which receives visual signals from the retina and transfers these visual information into the higher visual cortex. The most important pathology of RVO is obstruction of retinal veins, accompanied by hemorrhage and exudation and macular edema, which can lead to the retinal ganglion cell dysfunction. Thus, the visual loss of RVO might be contributed to the decreased ReHo values. Meanwhile, previous neuroimaging studies demonstrated that patients with RVO were associated with visual cortex dysfunction (Su et al., 2020). Consistent with these findings, our study found that patients with RVO showed a significant decrease in ReHo values in the bilateral visual cortex, which might be reflect the visual dysfunction in patients with RVO.

Another important finding is that patients with RVO showed increased ReHo values in the bilateral cerebellum_4_5 and decreased ReHo values in the right cerebellum_crus1.

The cerebellum is an important cerebral region, which responds to the controls of movement and balance perception. Previous neuroimaging studies have reported that cerebrovascular disease is associated with cerebellar dysfunction (Zhang et al., 2016; Koch et al., 2019). Dong et al. (2007) demonstrated that stroke patients showed fMRI activation in the perilesional primary motor cortex and cerebellum with training-related motor gains after stroke. Wei et al. (2020) found that ischemic pontine stroke patients showed significant gray matter volume (GMV) atrophy in the bilateral cerebellar posterior lobe. Thus, our study demonstrated that the patients with RVO showed abnormal ReHo values in the bilateral cerebellum, which might reflect that patients with RVO may have the same brain function changes as stroke.

Some limitations should be mentioned in the study. First, the sample size of this study is small. Second, in our study, patients with RVO were accompanied by hypertension and other diseases, which may have a certain impact on the results of the study. Third, during fMRI scanning, MRI machine noise and physiological noises (breathing and heart rate) can affect BOLD signals.

## Conclusion

Our study demonstrated that patients with RVO were associated with abnormal synchronous neural activities in the cerebellum, middle occipital gyrus, and inferior temporal gyrus. These findings shed new insight into neural mechanisms of vision loss in patients with RVO.

## Data availability statement

The raw data supporting the conclusions of this article will be made available by the authors, without undue reservation.

## Ethics statement

The studies involving human participants were reviewed and approved by the Ethical Committee for Medicine of Jiangxi Provincial People’s Hospital. The patients/participants provided their written informed consent to participate in this study. Written informed consent was not obtained from the individual(s) for the publication of any potentially identifiable images or data included in this article.

## Author contributions

YX, FG, HL, and YZ contributed to data collection, statistical analyses, wrote the manuscript, designed the protocol, contributed to the MRI analysis, designed the study, oversaw all clinical aspects of study conduct, and manuscript preparation. All authors contributed to the article and approved the submitted version.

## Footnotes

1
http://www.rfmri.org/dpabi
